# Effects of neuraxial labor analgesia on intrapartum maternal fever in full-term pregnancy and its influence on birth outcomes

**DOI:** 10.3389/fmed.2023.1208570

**Published:** 2023-07-18

**Authors:** Zhen Zhang, Chun-Mei Deng, Jia-Hui Ma, Shu Li, Bo Lei, Ting Ding

**Affiliations:** ^1^Department of Anesthesiology, Peking University First Hospital, Beijing, China; ^2^Department of Anesthesiology, Beijing Obstetrics and Gynecology Hospital, Beijing, China; ^3^Department of Anesthesiology, Haidian Maternal and Child Health Hospital, Beijing, China

**Keywords:** neuraxial labor analgesia, intrapartum fever, perinatal outcomes, full-term pregnancy, perinatal period

## Abstract

**Introduction:**

This study aimed to explore the relationship between neuraxial labor analgesia and intrapartum fever and to demonstrate the influence of maternal fever on perinatal outcomes within 6 weeks after birth.

**Methods:**

This was a secondary analysis of a multicenter prospective cohort study that enrolled women with single- and full-term cephalic pregnancy in northern China. Intrapartum maternal fever was defined as the highest axillary temperature during labor ≥37.5°C. Data on baseline characteristics, maternal variables, and neonatal outcomes were all collected. The association between neuraxial labor analgesia and intrapartum maternal fever was analyzed with logistic regression models, and the cutoff point was identified by the receiver operating characteristic curve.

**Results:**

Of 577 parturients, 74 (12.8%) developed intrapartum fever. Neuraxial analgesia was associated with an increased risk of maternal intrapartum fever with or without adjusting for confounding factors (adjusted OR = 2.68; 95% CI: 1.32–5.47; *p* = 0.007). Further analysis showed that neuraxial analgesia of <5 h did not increase the risk of intrapartum fever compared with no analgesia (OR = 1.52; 95% CI: 0.63–3.64; *p* = 0.35), and longer neuraxial labor analgesia time (over 5 h) significantly increased the risk of fever (OR = 3.38; 95% CI: 1.63–7.01; *p* = 0.001). Parturients with intrapartum fever suffered more maternal adverse outcomes compared with those without fever (*p* **<** 0.001). Neonates of women with intrapartum fever had slightly higher rates of composite adverse neonatal outcomes compared with those without fever; however, the difference was not statistically significant (*p* = 0.098).

**Conclusion:**

In women with low-risk pregnancies, a longer time of neuraxial labor analgesia was associated with an increased risk of intrapartum maternal fever. Intrapartum fever was related to adverse maternal outcomes but did not significantly affect neonatal outcomes within 6 weeks after delivery.

## Introduction

Intrapartum maternal fever affects up to one-third of all labors, which might be associated with increased risks of maternal complications, such as intrauterine infection, dystocia, and emergency cesarean delivery (1–3). Moreover, it is also reported to be a potential risk factor for adverse neonatal outcomes, including low Apgar scores, respiratory distress, hypotonia, neonatal brain injury, and even cerebral palsy (4). Multiple obstetric factors, such as intrapartum infection, premature rupture of membranes, Group B streptococcus positive, and prolonged duration of labor, might be involved in the process of maternal fever during labor (5). Moreover, it has been hypothesized that neuraxial analgesia (i.e., epidural-related maternal fever, ERMF) might play a role in intrapartum maternal fever (6, 7).

Neuraxial labor analgesia, including epidural analgesia and combined spinal–epidural analgesia, has been widely used as an effective method to relieve labor pain for decades (8). Many studies have investigated the association between neuraxial labor analgesia and intrapartum maternal fever in the past few years. A randomized clinical trial conducted in Brazil reported that the use of combined spinal and epidural anesthesia was associated with a significant increase in maternal temperature during vaginal delivery (9). The result of a recently published prospective observational study also showed significant associations between epidural labor analgesia and intrapartum maternal fever in all stages of labor (2). However, negative results were also reported (10). Considering the potential adverse effect of intrapartum maternal fever on perinatal outcomes (3), more investigations are still needed to provide further evidence on this issue. The objective of this study was to explore the association between neuraxial labor analgesia and intrapartum maternal fever and to further demonstrate the influence of maternal fever on birth outcomes in low-risk populations of full-term pregnant women.

## Materials and methods

### Ethics approval

The study protocol was approved by the Clinical Research Ethics Committees in Peking University First Hospital (2014 [714]) and the institutional review boards of other participating centers and was registered with the Chinese Clinical Trial Registry (www.chictr.org.cn; ChiCTR-OCH-14004888) and ClinicalTrials.gov (NCT02823418). Written informed consent was obtained from all participants before enrollment.

### Study design and participants

This study was a secondary analysis of multicenter prospective cohort research in northern China, which was conducted from 1 August 2014 to 29 May 2015 in Peking University First Hospital (a tertiary general hospital), Beijing Obstetrics and Gynecology Hospital (a tertiary specialized hospital) and Haidian Maternal and Child Health Hospital (a secondary specialized hospital) in Beijing, China. Detailed inclusion and exclusion criteria of the participants have been described previously (11). In brief, pregnant women were eligible for inclusion if they were nulliparae with full-term single-term cephalic pregnancy (>7 weeks) and prepared for vaginal delivery, who were clinically considered as women with low-risk pregnancies. Patients were excluded if they were younger than 18 years or older than 35 years, had a history of psychiatric disease, had contraindications to neuraxial analgesia, or were admitted to the delivery room outside the daytime working hours (from 5 p.m. to next 8 a.m.).

### Procedures

In this study, the decision of whether to receive neuraxial labor analgesia or not was made by the parturients themselves. As a clinical routine, 40 mg methylprednisolone was administered to parturients *via* intravenous infusion or bolus injection before lumber puncture in the participating medical centers to prevent nausea and vomiting. Epidural analgesia or combined spinal–epidural analgesia was performed on women who requested neuraxial analgesia when the cervix was dilated to 1 cm or more. For epidural analgesia, a loading dose of 10 ml mixture (0.1% ropivacaine and 0.5 μg/ml sufentanil) was administered through the epidural catheter. An additional dose of 5 ml mixture was administered 10 min later if the numeric rating scale (NRS, an 11-point scale where 0 = no pain and 10 = the worst pain) pain score remained at least 4. A patient-controlled epidural analgesia (PCEA) pump was connected 30 min later, which was established with a mixture of 0.1% ropivacaine (AstraZeneca AB, Södertälje, Sweden) plus 0.5 μg/ml sufentanil (EuroCept BV, Ankeveen, Netherlands) and programmed to deliver a 6 ml bolus with a 15-min lockout interval. For combined spinal–epidural analgesia, 2 to 3 ml of 0.1% ropivacaine was administered intrathecally. A PCEA pump was connected later, which was established with a mixture of 0.1% ropivacaine and 0.5 μg/ml sufentanil, programmed to deliver a 5 ml bolus with a 15-min lockout interval and a 5 m/h background infusion. Obstetric management during labor, such as oxytocin administration, intramuscular meperidine administration, forceps assistance, and cesarean delivery, were decided by the obstetricians as clinical routine.

### Data collection

For all recruited subjects, data on baseline characteristics, maternal variables, and neonatal variables were collected. The detail shows as follows:

(1) Baseline characteristics included sociodemographic information, previous medical history, and obstetrical data of the present pregnancy.

(2) Maternal variables included the use of neuraxial labor analgesia, medications during labor (including oxytocin, meperidine, and antibiotic administration), durations of all stages of labor, body temperature during labor (including baseline temperature, highest temperature, and postpartum temperature), mode of delivery, and estimated blood loss.

(3) Neonatal variables included gender, birthweight, Apgar Scores at 1 and 5 min after delivery, the occurrence of intrauterine fetal distress, assisted ventilation, and admission to the neonatal ward within 1 day postpartum. We also recorded the mode of infant feeding and other health-related problems through a face-to-face follow-up on the first day after delivery and a telephone interview at 6 weeks postpartum.

The occurrence of intrapartum maternal fever was defined as the highest axillary temperature during labor more than or equal to 37.5°C. Axillary temperature was measured by midwives with mercury thermometers every 2 h or when considered necessary since delivery room admission. Baseline temperature and postpartum temperature were defined as the temperature on admission to the delivery room and 2 h after delivery, respectively.

### Statistical analysis

Data were summarized as mean ± standard deviation, number (proportions), or median (interquartile range). The independent *t*-test, Mann–Whitney U-test, and chi-squared (χ2) test were used to test statistical significance between groups of continuous (normal and non-normal distribution data) and categorical variables, respectively. A logistic regression model was applied to determine the association between neuraxial labor analgesia and intrapartum maternal fever. Variables that showed significant differences between the two groups (*p* < 0.05) as well as variables that might potentially affect intrapartum temperature based on clinical grounds were adjusted in the above model. Odds ratios (ORs) and 95% confidence intervals (95% CIs) were estimated. The receiver operating characteristic (ROC) curve was used to identify the cutoff point of the association between the duration time of neuraxial labor analgesia and intrapartum fever. Exploratory analyses were performed to assess differences in the primary outcome in subgroups. Treatment-by-covariate interactions were assessed separately for each subgroup factor using logistic regression. Two-tailed *p*-values of < 0.05 were considered statistically significant. Statistical analyses were performed using SPSS version 22.0 (IBM SPSS Inc., Chicago, IL, USA).

## Results

### Patient recruitment and baseline characteristics

**Figure 1:**
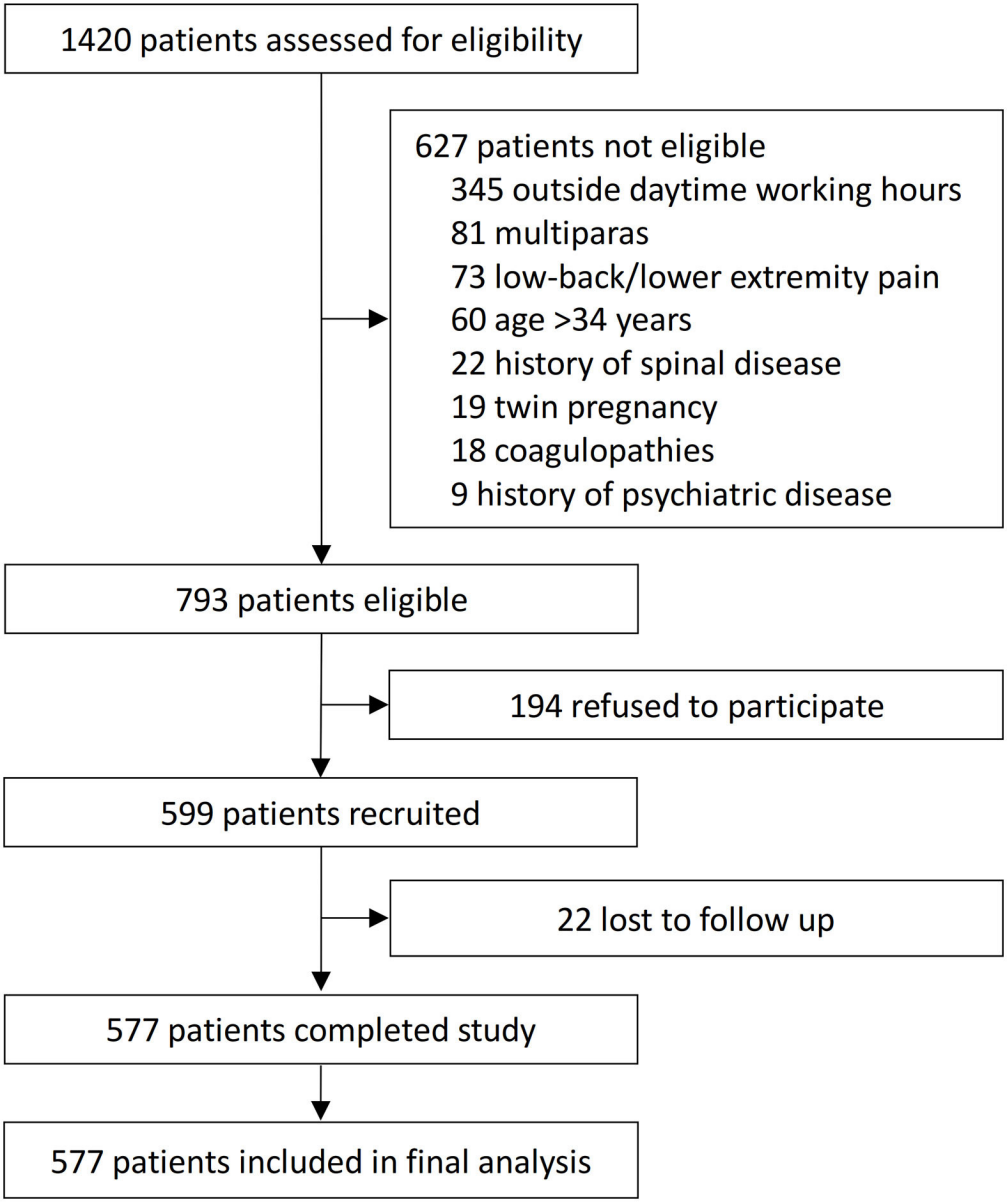
Flowchart of the study.

In total, 1,420 parturients were screened; of these, 793 were eligible, 599 were enrolled and 577 completed the study (Figure 1). There were no significant differences in baseline information between enrolled and not enrolled patients (Supplementary Table S1). All 577 parturients were included in this study with an age range between 22 and 34 years. Of these, 74 (12.8%) developed intrapartum fever (cases), and 503 (87.2%) did not develop a fever (controls). The baseline variables of all parturients are shown in Table 1. There were no significant differences between the two groups with regard to sociodemographic characteristics, medical comorbidity, and obstetrical data. The incidence of fever was higher among people without gynecological diseases (*p* = 0.035).

**Table 1:** Baseline characteristics of parturients with and without fever.

	**All parturients (*n* = 577)**	**Fever (*n* = 74)**	**No fever (*n* = 503)**	***p*-value**
Maternal age (year)	29.9 ± 2.5	29.9 ± 2.3	30.0 ± 2.6	.764
Body mass index before childbirth (kg/m^2^)				.298
< 30	484 (83.9%)	59 (79.7%)	425 (84.5%)	
≥30	93 (16.1%)	15 (20.3%)	78 (15.5%)	
Han nationality^a^	545 (94.5%)	67 (90.5% )	478 (95.0%)	.192
Medical comorbidity^c^	42 (7.3%)	4 (5.4%)	38 (7.6%)	.506
Gynecological diseases^d^	54 (9.4%)	2 (2.7%)	52 (10.3%)	.035
Surgery history	83 (14.4%)	8 (10.8%)	75 (14.9%)	.348
Adverse pregnancy history^e^	188 (32.6%)	21 (28.4%)	167 (33.2%)	.409
Dysmenorrhea	317 (54.9%)	38 (51.4%)	279 (55.5%)	.506
Obstetric complications				
Diabetes^f^	130 (22.5%)	14 (18.9%)	116 (23.1%)	.426
Hypertensive disorders^g^	34 (5.9%)	3 (4.1%)	31 (6.2%)	.472
Hypothyroidism	43 (7.5%)	3 (4.1%)	40 (8.0%)	.233
Anemia^h^	60 (10.4%)	7 (9.5%)	53 (10.5%)	.777
Prepartum hemoglobin (g/l)	12.4 ± 1.2	12.4 ± 1.3	12.4 ± 1.1	.682
PROM^i^	108 (18.7%)	18 (24.3%)	90 (17.9%)	.185
Duration of gestation (day)	277 ± 7	278 ± 7	277 ± 7	.509
Labor description				.320
Spontaneous	392 (67.9%)	54 (73.0%)	338 (67.2%)	
Induction	185 (32.1%)	20 (27.0%)	165 (32.8%)	

Data are presented as mean ± SD, number (%).

a Other nationalities included Manchu, Mongol, Huis, Koreans and Yi.

^b^ Included Buddhism, Islam and Christianity.

c Included asthma, arrhythmia, latent glomerulonephritis, abnormal liver function and positive hepatitis B surface antigen.

d Included hysteromyoma, ovarian cysts, dysfunctional uterine bleeding, polycystic ovary syndrome and pelvic inflammatory disease.

e Included history of miscarriages, induced abortion, and midtrimester induction of labor due to fetal anomalies.

f Included both impaired glucose tolerance and gestational diabetes mellitus (GDM) diagnosed by the obstetrics.

g Included preeclampsia-eclampsia, chronic hypertension, chronic hypertension with superimposed preeclampsia and gestational hypertension according to the American College of Obstetrics and Gynecology (ACOG) guideline.

h The concentration of hemoglobin lower than 110 g/l.

i Premature rupture of membranes.

### Labor and delivery characteristics

Parturients with intrapartum fever had higher rates of neuraxial labor analgesia (*p* = 0.003) and intrapartum antibiotic administration (*p* < 0.001), had longer durations of the first and second labor stages (*p* < 0.001 and *p* = 0.011, respectively), developed higher postpartum temperature **(***p* = 0.001**)**, and underwent more lateral episiotomy (*p* = 0.011). The rates of instrumental and cesarean deliveries were significantly higher in women who suffered from intrapartum fever (*p* < 0.001). Moreover, patients with fever had more blood loss during labor (*p* = 0.029) and longer lengths of postpartum hospital stay (*p* = 0.004) compared with those without fever. Overall, the prevalence of composite adverse maternal outcomes was almost 2-fold in the fever group (59.5% vs. 35.0%, *p* < 0.001). In addition, the length of postpartum hospital stay was significantly longer in parturients with intrapartum fever than those without (*p* = 0.004). All the detailed information on perinatal maternal variables is shown in Table 2.

**Table 2:** Labor and delivery characteristics of parturients with and without fever.

	**All parturients (*n* = 577)**	**Fever (*n* = 74)**	**No fever (*n* = 503)**	***p*-value**
Neuraxial analgesia	417 (72.3%)	64 (86.5%)	353 (70.2%)	.003
Epidural	272 (65.2%)	42 (65.6%)	230 (65.2%)	
Combined spinal-epidural	145 (34.8%)	22 (34.4%)	123 (34.8%)	
**Duration of labor**				
First stage (min)^a^	550 (360–780)	730 (510–998)	540 (340–750)	**< .001**
Second stage (min)^a^	46 (28–79)	67 (39-103)	45 (27–75)	**.011**
Third stage (min)^a^	7 (5–10)	6 (5–10)	7 (5–10)	.967
Intrapartum oxytocin administration	384 (66.6%)	46 (62.2%)	338 (67.2%)	.391
Intrapartum meperidine administration^b^	14 (2.4%)	1 (1.4%)	13 (2.6%)	.811
Intrapartum antibiotic administration	281 (48.7%)	66 (89.2%)	215 (42.7%)	**< .001**
Artificial rupture of membranes	215 (37.3%)	25 (33.8%)	190 (37.8%)	.507
Baseline temperature^c^	36.7 ± 0.3	36.7 ± 0.3	36.7 ± 0.3	**.216**
Maximum recorded temperature in labor	37.0 ± 0.4	37.7 ± 0.3	37.0 ± 0.3	**< .001**
Postpartum temperature^d^	37.0 ± 0.3	37.1 ± 0.3	37.0 ± 0.3	**.001**
**Mode of delivery**				**.001**
Spontaneous delivery	382 (66.2%)	35 (47.3%)	347 (69%)	
Cesarean delivery	137 (23.7%)	27 (36.5%)	110 (21.9%)	
instrumental delivery	58 (10.1%)	12 (16.2%)	46 (9.1%)	
Lateral episiotomy^e^	186 (42.3%)	28 (59.6%)	158 (40.2%)	.011
Estimated blood loss (ml)	200 (200–300)	300 (200–400)	200 (150–300)	.029
Postpartum hemorrhage^f^	39 (6.8%)	7 (9.5%)	32 (6.4%)	.322
Exclusive breast-feeding 1-day postpartum	465 (80.6%)	60 (81.1%)	405 (80.5%)	.909
Composite adverse maternal outcome^g^	220 (38.1%)	44 (59.5%)	176 (35.0%)	**< .001**
Length of postpartum hospital stay (days)	2 (2–4)	3 (2–4)	2 (2–4)	**.004**

Data are presented as mean ± SD, number (%) or median (interquartile range).

a Excluded 137 patients (27 with intrapartum maternal fever and 110 without) who underwent emergency cesarean delivery during the first stage of labor.

b Meperidine 100 mg was administered intramuscularly according to the prescription of obstetricians.

c Temperature at the beginning of labor.

d Temperature 2 h postpartum.

e Results of parturients who gave spontaneous or forceps delivery.

f Defined as blood loss of more than 500 mL after vaginal delivery or 1,000 mL after cesarean section within 24 h following delivery.

g Defined as any of the followings: instrumental delivery, cesarean delivery or post-partum hemorrhage.

*P* values in bold indicates those < 0.05.

### Association between neuraxial labor analgesia and intrapartum maternal fever

Based on logistic regression models, a univariate analysis was used to demonstrate the association between neuraxial labor analgesia and intrapartum maternal fever; six potential confounders (i.e., gynecological diseases before pregnancy, obstetric complications, premature rupture of membranes, baseline temperature, intrapartum meperidine administration, and birthweight of newborns) were adjusted in the adjusted logistic regression model. The result showed that intrapartum maternal fever was associated with neuraxial labor analgesia with or without adjustment for potential confounders (adjusted OR = 2.68; 95% CI: 1.32–5.47; *p* = 0.007) (Table 3).

**Table 3:** Associations between neuraxial labor analgesia and intrapartum maternal fever.

	**Fever/total (n/N)**	**Crude OR (95% CI)**	***p*-value**	**Adjusted OR (95% CI) ^a^**	***p*-value**
**Neuraxial labor analgesia**				
No	10/160	Reference	Reference	Reference	Reference
Yes	64/417	2.72 (1.36–5.44)	**.005**	2.68 (1.32–5.47)	**.007**
**Duration of neuraxial labor analgesia**				
No neuraxial analgesia	10/160	Reference	Reference	Reference	Reference
< 5 h	13/149	1.43 (0.61–3.38)	.41	1.52 (0.63–3.64)	.35
≥5 h	51/268	3.53 (1.74–7.16)	**< .001**	3.38 (1.63-7.01)	**.001**

OR, odds ratio; CI, confidence interval.

a Adjusted for gynecological diseases before pregnancy, obstetric complications, PROM, baseline temperature, intrapartum meperidine administration and birthweight of newborns.

*P* values in bold indicates those < 0.05.

ROC curve was applied to investigate the cutoff points of the association between the duration time of neuraxial labor analgesia and intrapartum fever. The result suggested that 5 h was the cutoff point (Supplementary Figure S1). As the duration of neuraxial labor analgesia increased, the risk of intrapartum maternal fever increased. Compared with those parturients without neuraxial labor analgesia, the risk of intrapartum maternal fever in parturients with shorter neuraxial labor analgesia time (< 5 h) and parturients with longer neuraxial labor analgesia time (over 5 h) increased by 52% (OR = 1.52; 95% CI: 0.63–3.64; *p* = 0.35) and 238.0% (OR = 3.38; 95% CI: 1.63–7.01; *p* = 0.001), respectively. All of the above results are shown in Table 3.

**Figure 2:**
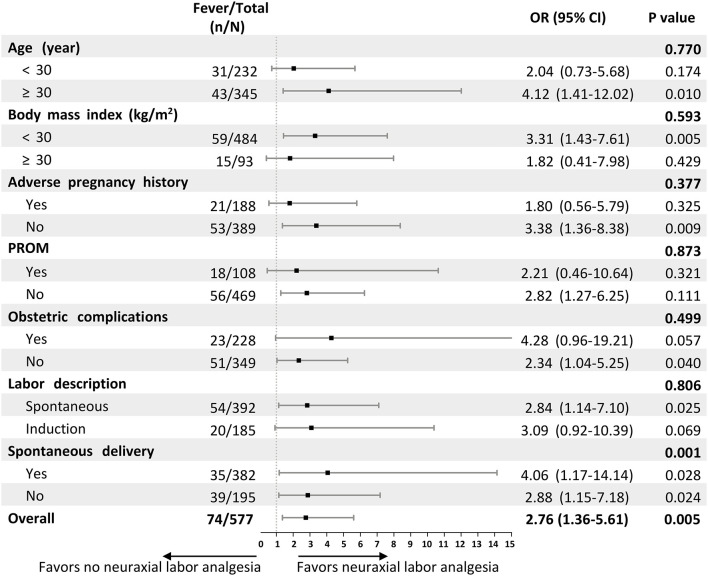
Forest plot assessing interactions between subgroups and the associations between neuraxial labor analgesia and intrapartum maternal fever. ORs and interim-adjusted 95% CIs are shown. The estimated overall OR was derived from a logistic regression model adjusted for the confounder variable in this study including gynecological diseases before pregnancy, obstetric complications, PROM, baseline temperature, and intrapartum meperidine administration. For the subgroup analyses, we assessed the treatment-by-covariate interaction on the association between neuraxial labor analgesia and intrapartum maternal fever, adjusting for the same variables. OR, Odds ratio; CI, confidence interval; PROM, premature rupture of membrane.

Subgroup analyses of factors related to intrapartum maternal fever were performed to evaluate the consistency of the effect of neuraxial labor analgesia on intrapartum maternal fever (Figure 2). The results of the subgroup analysis did not show the heterogeneity of risk of incident intrapartum maternal fever from neuraxial labor analgesia besides the subgroup of parturients whether spontaneous delivery or not.

### Neonatal outcomes

For neonatal information, the birthweight of a newborn was significantly heavier in parturients with intrapartum fever (*p* = 0.016) (Table 4). There were no significant differences in rates of fetal distress, low 1 and 5 min Apgar scores, ventilation support, neonatal ward admission, neonatal infection, and readmission to hospital within 6 weeks after birth between the two groups. In total, the proportion of composite adverse neonatal outcomes was slightly higher in infants with febrile mothers, although the difference was not statistically significant (15 [20.3%] vs. 66 [13.1%], *p* = 0.098) (Table 4).

**Table 4:** Neonatal variables of parturients with and without fever.

	**All parturients (*n* = 577)**	**Fever (*n* = 74)**	**No fever (*n* = 503)**	***p*-value**
Male	317 (54.9%)	46 (62.2%)	271 (53.9%)	.181
Birthweight (g)	3419 ± 396	3530 ± 417	3402 ± 391	**.016**
Fetal distress^a^	83 (14.4%)	15 (20.3%)	68 (13.5%)	.122
1-min Apgar score	10 (10)	10 (10)	10 (10)	.407
1-min Apgar score < 7	11 (1.9%)	0 (0%)	11 (2.2%)	.199
5-min Apgar score	10 (8–10)	10 (10)	10 (10)	.466
5-min Apgar score < 7	4 (0.7%)	0 (0%)	4 (0.8%)	.984
Immediate need for assisted ventilation	47 (8.1%)	8 (10.8%)	39 (7.8%)	.369
Neonatal ward admission within 1-day after birth^b^	56 (9.7%)	10 (13.5%)	46 (9.1%)	.236
Neonatal infection	27 (4.7%)	6 (8.1%)	21 (4.2%)	.135
Hemodynamic instability	0 (0%)	0 (0%)	0 (0%)	.999
Seizures	0 (0%)	0 (0%)	0 (0%)	.999
Composite adverse neonatal outcome^c^	81 (14.0)	15 (20.3%)	66 (13.1%)	.098
**6-Week postpartum**				
Exclusive breast-feeding	397 (68.8%)	49 (66.2%)	348 (69.2%)	.607
Baby readmitted to hospital	9 (1.6%)	1 (1.4%)	8 (1.6%)	.999

Data are presented as mean ± SD, number (%) or median (interquartile range).

a Fetal distress was considered when category III tracing or repeated category II tracing was presented by electronic fetal monitoring during labor.

b Newborns were admitted to neonatal ward for further monitoring and/or treatment which were considered necessary by the pediatricians.

c Defined as any of followings: assisted ventilation, 1-min Apgar score < 7, 5-min Apgar score < 7, or neonatal ward admission within 1-day after birth.

*P* values in bold indicates those < 0.05.

## Discussion

Our study demonstrated that, among young nulliparous women with single and full-term cephalic pregnancy, the use of neuraxial analgesia during labor, especially duration of neuraxial analgesia of more than 5 h, was associated with an increased risk of intrapartum fever. Moreover, intrapartum fever was related to maternal adverse outcomes but did not significantly affect the short-term outcomes of neonates.

Maternal intrapartum fever, also called intrapartum hyperthermia, is commonly defined as a temperature more than 37.5°C or 38°C during labor in previous studies (3). The incidence of intrapartum hyperthermia has varied strikingly from 1 to 37% due to different populations and diagnostic criteria (12, 13). Although 38°C was used mostly in previous studies as the definition of maternal fever, we still used 37.5°C as the threshold of clinical fever because the association between low-grade fever (≥37.5°C) and adverse neonatal outcomes has been previously reported (13, 14). It is worth mentioning that the incidence of maternal fever in our study was lower than in other works of literature among Chinese women (15). It is possible that the relatively low concentration of neuroblockade we used for neuraxial analgesia and also different obstetric practices, such as methylprednisolone, meperidine, and antibiotic administrations, might be involved in the lower incidence of intrapartum fever. Goetzl et al. reported that the administration of high-dose corticosteroids during labor resulted in a 90% reduction of maternal fever in parturients with epidural analgesia, suggesting that steroids might decrease the risk of epidural intrapartum fever (16). Thus, we speculated that intrapartum steroid treatment of parturients in this study might be associated with the reduction of intrapartum fever to some extent, and the underlying relationship needs to be further explored.

Our results showed that in women with low-risk pregnancies, neuraxial analgesia was significantly associated with an increased incidence of mild maternal fever, which was in line with previous studies (2, 15). In fact, the etiology of epidural-related maternal fever remains elusive and the sterile inflammation process was considered to be a possible mechanism (17). A randomized controlled trial with prophylactic antibiotics before epidural analgesia showed no difference in the rate of maternal fever between groups, supporting the non-infectious inflammation hypothesis of epidural fever (18). A study reported that acetaminophen prophylaxis did not prevent maternal hyperthermia or fever secondary to epidural analgesia, also suggesting a non-infectious inflammatory process (19). In addition, local anesthetics used in epidural were also reported to trigger non-infectious inflammation pathways *via* immunomodulation and cell injury (20, 21). Overall, non-infectious inflammation might be involved in the ERMF and the exact mechanisms need to be further clarified.

Our study also found that the risk of intrapartum fever increased significantly in parturients with neuraxial labor analgesia time of more than 5 h. A recently published observational study also reported weak time and dose-dependent correlations between PCEA and intrapartum fever, and an analgesic time over 6.3 h increased the risk of maternal intrapartum fever (22). However, inconsistent results also existed. Wang et al. found that although the duration of analgesia in the early PCEA group was significantly longer than that in the late PCEA group, the average maternal temperature and the incidence of fever were not significantly different, suggesting that the duration of epidural analgesia might not be an important determinant of epidural-related fever (23, 24). Further research is still needed to further clarify the time and dose-dependent correlations.

Numerous studies have demonstrated that intrapartum hyperthermia was associated with a series of adverse obstetric outcomes in mothers. Dior et al. found that maternal fever was significantly associated with adverse maternal outcomes, including postpartum hemorrhage, labor dystocia, and cesarean section (25). The study of Lange et al. also reported that parturients who developed fever were more likely to have prolonged durations of labor and required cesarean delivery (26). Results of this study showed that febrile parturients underwent more cesarean section or instrumental delivery, suffered more postpartum blood loss, and had a longer length of postpartum hospital stay, further demonstrating that intrapartum fever was associated with adverse maternal outcomes. It is possible that the elevation of body temperature is an early indicator of potential obstetric abnormalities, and early attention and interventions should be taken to decrease the potential adverse events followed by intrapartum fever.

In this study, we evaluated neonatal outcomes of term infants and found no significant association between intrapartum maternal fever and adverse neonatal outcomes within 6 weeks after birth. However, the effects of intrapartum fever on newborns remain controversial and conflicting evidence existed in this field (13, 27, 28). A retrospective cohort study reported that adverse neonatal outcomes including assisted ventilation, Apgar scores <7, and NICU admission increased infants' exposure to maternal hyperthermia (1). A recently published meta-analysis including 41 studies suggested that maternal intrapartum fever of any cause was associated with neonatal brain injury (3). It is possible that the definition of maternal fever in this study was over 37.5°C, less than that of some other studies, resulting in fewer influences on intrauterine fetuses. In addition, the inclusion of low-risk parturients, who were young and with single-term pregnancies in our study, might lead to relatively low-risk infants. Moreover, obstetric and pediatric management in participating medical centers might influence the birth outcomes to some extent. Further studies with larger sample sizes and longer time of follow-ups are still needed to explore the long-term effect of maternal fever on neonatal outcomes.

There are strengths and also some limitations in this analysis. Intrapartum maternal fever is a prevalent occurrence, affecting approximately one-third of all labors, which is associated with an increased risk of maternal complications and has the potential to impact neonatal outcomes. However, the impact of epidural-related maternal fever on maternal and neonatal outcomes within 6 weeks following childbirth remains inconclusive. Our data contribute additional evidence on this issue, particularly focusing on primiparous women with low-risk and term pregnancies in the Asia population. Furthermore, many previous studies investigating perinatal outcomes of epidural analgesia were conducted without an opioid-free control group, which may have introduced interference when comparing results to those involving opioids. In this study, the use of opioids was relatively conservative, aligning with local clinical routine, and the proportion of administration of opioid drugs in the control group was very low. Therefore, our study offers insights into the effects of neuraxial analgesia, to some extent, without the confounding influence of opioids. Meanwhile, the study also has some limitations. First, the maternal temperature during labor was not continuously monitored, which might lead to a potential misdiagnose of intrapartum fever. Second, as an observational study, it is inevitable that unidentified confounding factors might influence the results. Although a multivariate regression model was adopted to adjust for potential confounders of maternal fever, we cannot neglect the possible interference of perinatal factors. Third, as routine screening for inflammatory parameters and placental pathology was not performed during hospitalization, data collection before and/or after the neuraxial procedures was not feasible. Fourth, the neonatal outcomes may not be comprehensive enough. Future studies should consider adding more neonatal parameters, such as fetal acidosis, antibiotic treatment, and admission to the neonatal intensive care unit in the neonatal outcome assessment. Finally, as a secondary analysis of a multicenter research database, the sample size might be not enough to establish a causal relationship.

Despite limitations, we found that in nulliparae with single- and full-term cephalic pregnancy, a longer time of neuraxial labor analgesia was associated with an increased risk of intrapartum maternal fever. Intrapartum fever was related to adverse maternal outcomes but did not significantly affect the neonatal outcomes of low-risk mothers within 6 weeks after delivery.

## Data availability statement

The original contributions presented in the study are included in the article/Supplementary material, further inquiries can be directed to the corresponding author.

## Ethics statement

The studies involving human participants were reviewed and approved by Clinical Research Ethics Committees in Peking University First Hospital. The patients/participants provided their written informed consent to participate in this study.

## Author contributions

ZZ and C-MD contributed to the study design, data collection, and manuscript writing. J-HM contributed to the study design, data analysis, and manuscript writing. SL and BL helped to recruit patients and collected the clinical data. TD contributed to the study design, manuscript writing, and revision. All authors read and approved the final manuscript.

## Supplementary material

Supplementary Figure S1 Receiver operating characteristic curve of the duration time of neuraxial labor analgesia and intrapartum fever (sensitivity = 0.813, specificity = 0.382, the area under the ROC = 0.577, and 95%CI: 0.504–0.650).
