# Supplementary material for: Effects of neuraxial labor analgesia on intrapartum maternal fever in full-term pregnancy and its influence on birth outcomes
Source: Front Med (Lausanne). 2023 Jul 18;10:1208570. doi: 10.3389/fmed.2023.1208570 (PMC10390729; doi:10.3389/fmed.2023.1208570)
Supplement: Supplementary file 1 [file Table_1.DOCX]

Supplement Table S1. Baseline variables of enrolled and not enrolled parturients.

|  | Eligible parturients  (n=793) | Not enrolled parturients  (n=194) | Enrolled parturients  (n=599) | *p* value |
| --- | --- | --- | --- | --- |
| Age (year) | 29.9±2.6 | 29.6±2.6 | 30.0±2.5 | .124 |
| Body mass index before childbirth (kg/m^2^) | 27.2±2.8 | 27.0±2.7 | 27.3±2.8 | .135 |
| Pregestational medical comorbidity ^a^ | 63 (7.9%) | 19 (9.8%) | 44 (7.3%) | .273 |
| Pregestational gynaecological disease ^b^ | 67 (8.4%) | 11 (5.7%) | 56 (9.3%) | .109 |
| History of abortion | 249 (31.4%) | 58 (29.9%) | 191 (31.9%) | .604 |
| History of surgery | 108 (13.6%) | 23 (11.9%) | 85 (14.2%) | .410 |
| Pregnancy with obstetric disease ^c^ | 243 (30.6%) | 53 (27.3%) | 190 (31.7%) | .248 |
| Duration of gestation (day) | 277±7 | 278±6 | 277±7 | .147 |
| Gravidity | 1 (1-5) | 1 (1-4) | 1 (1-5) | .567 |
| Received neuraxial labor analgesia | 575 (72.5%) | 139 (71.6%) | 436 (72.8%) | .758 |

Data are presented as mean ± SD, number (%) or median (interquartile range). ^a^ Include asthma, arrhythmia, latent glomerulonephritis, abnormal liver function and positive hepatitis B surface antigen. ^b^ Include hysteromyoma, ovarian cysts, dysfunctional uterine bleeding, polycystic ovary syndrome and pelvic inflammatory disease. ^c^ Include Impaired glucose tolerance, gestational diabetes mellitus, pregnancy-induced hypertension syndrome, preeclampsia and low free triiodothyronine and free thyroxine during pregnancy.
