# Supplementary figures and images for: Effects of neuraxial labor analgesia on intrapartum maternal fever in full-term pregnancy and its influence on birth outcomes
Source: Front Med (Lausanne). 2023 Jul 18;10:1208570. doi: 10.3389/fmed.2023.1208570 (PMC10390729; doi:10.3389/fmed.2023.1208570)

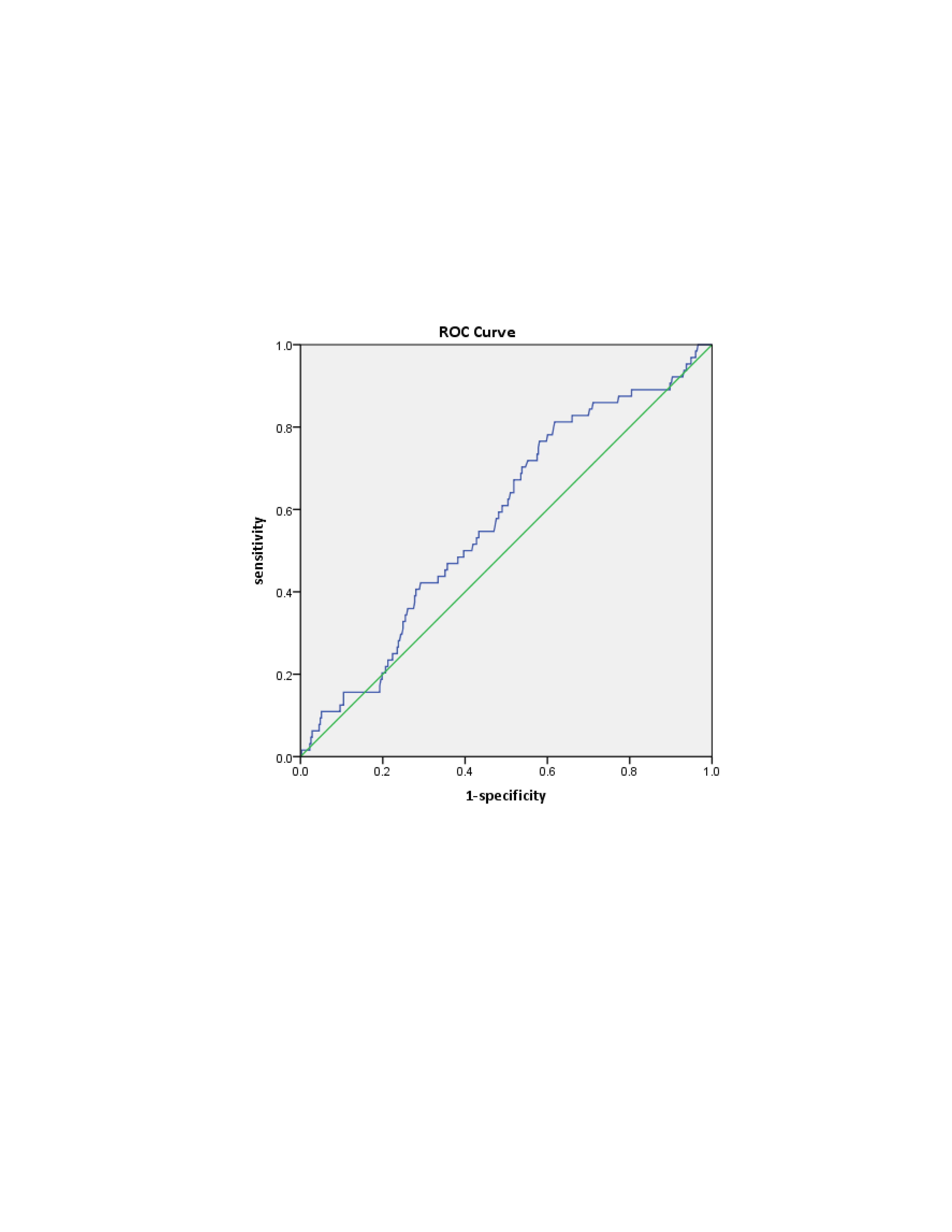

Supplement: Supplementary Figure S1 — Receiver operating characteristic curve of the duration time of neuraxial labor analgesia and intrapartum fever (sensitivity = 0.813, specificity = 0.382, the area under the ROC = 0.577, and 95%CI: 0.504–0.650). [file Image_1.TIF]
